# Red-Shifted Aequorin Variants Incorporating Non-Canonical Amino Acids: Applications in *In Vivo* Imaging

**DOI:** 10.1371/journal.pone.0158579

**Published:** 2016-07-01

**Authors:** Kristen M. Grinstead, Laura Rowe, Charles M. Ensor, Smita Joel, Pirouz Daftarian, Emre Dikici, Jean-Marc Zingg, Sylvia Daunert

**Affiliations:** 1 Department of Biochemistry and Molecular Biology, R. Bunn Gautier Bldg., 1011 NW 15th Street, Miller School of Medicine, University of Miami, Miami, FL, 33136, United States of America; 2 Department of Chemistry, 1610 Campus Drive East, Valparaiso University, Valparaiso, IN, 46385, United States of America; 3 Department of Pharmaceutical Sciences, College of Pharmacy, University of Kentucky, Lexington, KY, 40536, United States of America; Cardiff University, UNITED KINGDOM

## Abstract

The increased importance of *in vivo* diagnostics has posed new demands for imaging technologies. In that regard, there is a need for imaging molecules capable of expanding the applications of current state-of-the-art imaging *in vivo* diagnostics. To that end, there is a desire for new reporter molecules capable of providing strong signals, are non-toxic, and can be tailored to diagnose or monitor the progression of a number of diseases. Aequorin is a non-toxic photoprotein that can be used as a sensitive marker for bioluminescence *in vivo* imaging. The sensitivity of aequorin is due to the fact that bioluminescence is a rare phenomenon in nature and, therefore, it does not suffer from autofluorescence, which contributes to background emission. Emission of bioluminescence in the blue-region of the spectrum by aequorin only occurs when calcium, and its luciferin coelenterazine, are bound to the protein and trigger a biochemical reaction that results in light generation. It is this reaction that endows aequorin with unique characteristics, making it ideally suited for a number of applications in bioanalysis and imaging. Herein we report the site-specific incorporation of non-canonical or non-natural amino acids and several coelenterazine analogues, resulting in a catalog of 72 cysteine-free, aequorin variants which expand the potential applications of these photoproteins by providing several red-shifted mutants better suited to use *in vivo*. *In vivo* studies in mouse models using the transparent tissue of the eye confirmed the activity of the aequorin variants incorporating L-4-iodophehylalanine and L-4-methoxyphenylalanine after injection into the eye and topical addition of coelenterazine. The signal also remained localized within the eye. This is the first time that aequorin variants incorporating non-canonical amino acids have shown to be active *in vivo* and useful as reporters in bioluminescence imaging.

## Introduction

Imaging is a critical component of medical diagnostics and biomedical research. X-Ray computed tomography, ultrasonography, magnetic resonance imaging (MRI), and positron emission tomography imaging (PET), and optical methods based on fluorescence or bioluminescence are used as stand-alone or in-tandem in diagnostic imaging to provide information on morphological, anatomical, or organ function. Despite their wide use, the majority of these methods do not have the ability to monitor specific molecular events linked to disease states and provide a complete picture of a biochemical process. *In vivo* imaging systems (IVIS) [1–4] is currently the state-of-the-art instrumentation used in molecular imaging to investigate biological processes in whole animal bioluminescence imaging (BLI) employing reporter technologies. IVIS has a fast image acquisition time (from seconds to a few minutes) and can be used to image several animals at once, providing a high throughput cost-effective alternative.

“Living light” or bioluminescence, is a naturally occurring phenomenon where light is generated by living organisms. Bioluminescence has several advantages over fluorescence, most notably the high sensitivity shown by the subattomole detection limits *in vitro*, and low background caused by the lack of inherent bioluminescence in most tissues [5–8]. Furthermore, bioluminescence does not require external illumination, making it suitable for light sensitive organs and tissues such as the retina.

Bioluminescent studies have frequently been conducted using the intracellular expression of proteins such as firefly luciferase (FLuc) and *Renilla* luciferase (RLuc) [9–21]. Though these proteins have the benefits of bioluminescence and have been used for *in vivo* cell tracking and gene expression, they require the incorporation of foreign DNA or the transplant of transgenic tissue [22, 23]. To overcome these drawbacks, we propose the direct administration of engineered red-shifted emission variants of the photoprotein aequorin, a protein employed previously in deep tissue calcium measurements [24].

**Fig 1:**
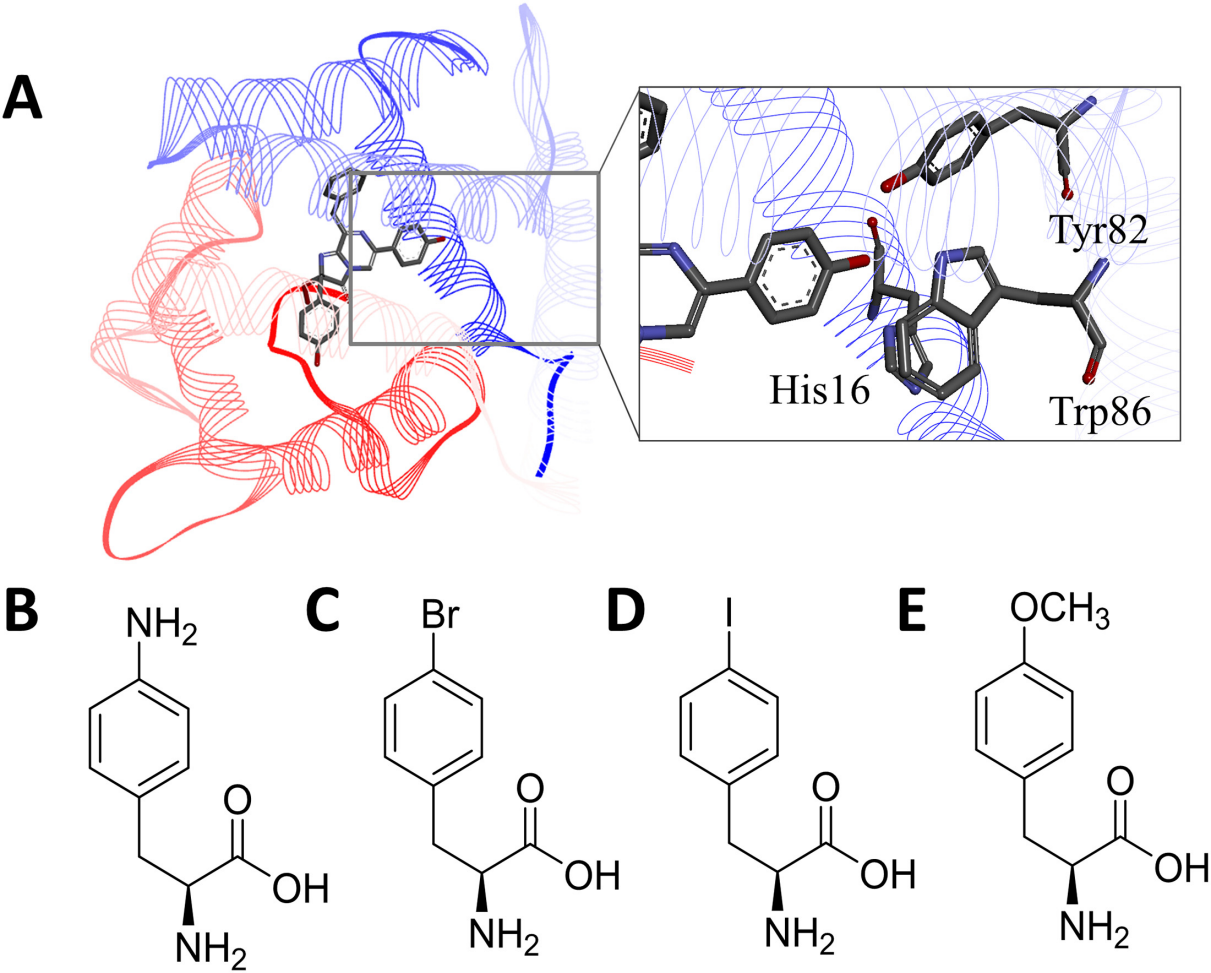
Structures of wild-type aequorin and the non-natural amino acid analogs employed for site-selective incorporation into the protein. (A) Crystal structure of aequorin with the location of the Tyr82-His16-Trp86. The substituted tyrosine is at position 82 and the substituted tryptophan is at position 86. (B) Chemical structure of non-natural amino acids: L-4-aminophenylalanine, (C) L-4-bromophenylalanine, (D) L-4-iodophenylalanine, (E) L-4 methoxyphenylalanine (PDB ID: 1EJ3). Reprinted from [47]under a CC BY license, with permission from *Open Access Dissertations*, original copyright 2015.

Aequorin is a 22 kDa non-toxic photoprotein originally isolated from the bioluminescent jellyfish *Aequorea victoria* and employed initially for *in vivo* calcium detection [25, 26]. In addition, aequorin has found numerous applications in cell trafficking [27, 28], biosensing [29–32], and in bioluminescent competitive [30] and immunoassays [33–41]. Aequorin uses the conversion of the incorporated luciferin coelenterazine to coelenteramide to emit a flash of light at 472 nm. The coelenterazine is bracketed in the hydrophobic pocket by H-bonds with three triads: Tyr184-His169-Trp173, Tyr192-His58-Trp108, and Tyr82-His16-Trp86 [42–44] (Fig 1). The addition of calcium causes a change in the conformation of the protein, triggering the bioluminescence reaction. The availability of different synthetic coelenterazine analogs with a variety of emission maxima and half-lives, allows for the tuning of emitted light [45, 46]. Since wild-type aequorin emits in the blue region of the visible spectrum, and since the emitted light at that particular wavelength is more easily absorbed and scattered by tissue, the availability of red-shifted aequorin mutants is important for further expanding its role *in vivo*.

In addition to synthetic coelenterazine, random and site-directed mutations have been used by us and others to create a variety of aequorin analogs with different wavelengths of emission and half-lives [8, 46, 48–54]. Although aequorin variants with unique spectral characteristics using canonical amino acids have been created, the range of emission properties can be further tailored by strategic incorporation of non-natural amino acids [46, 53].

**Fig 2:**
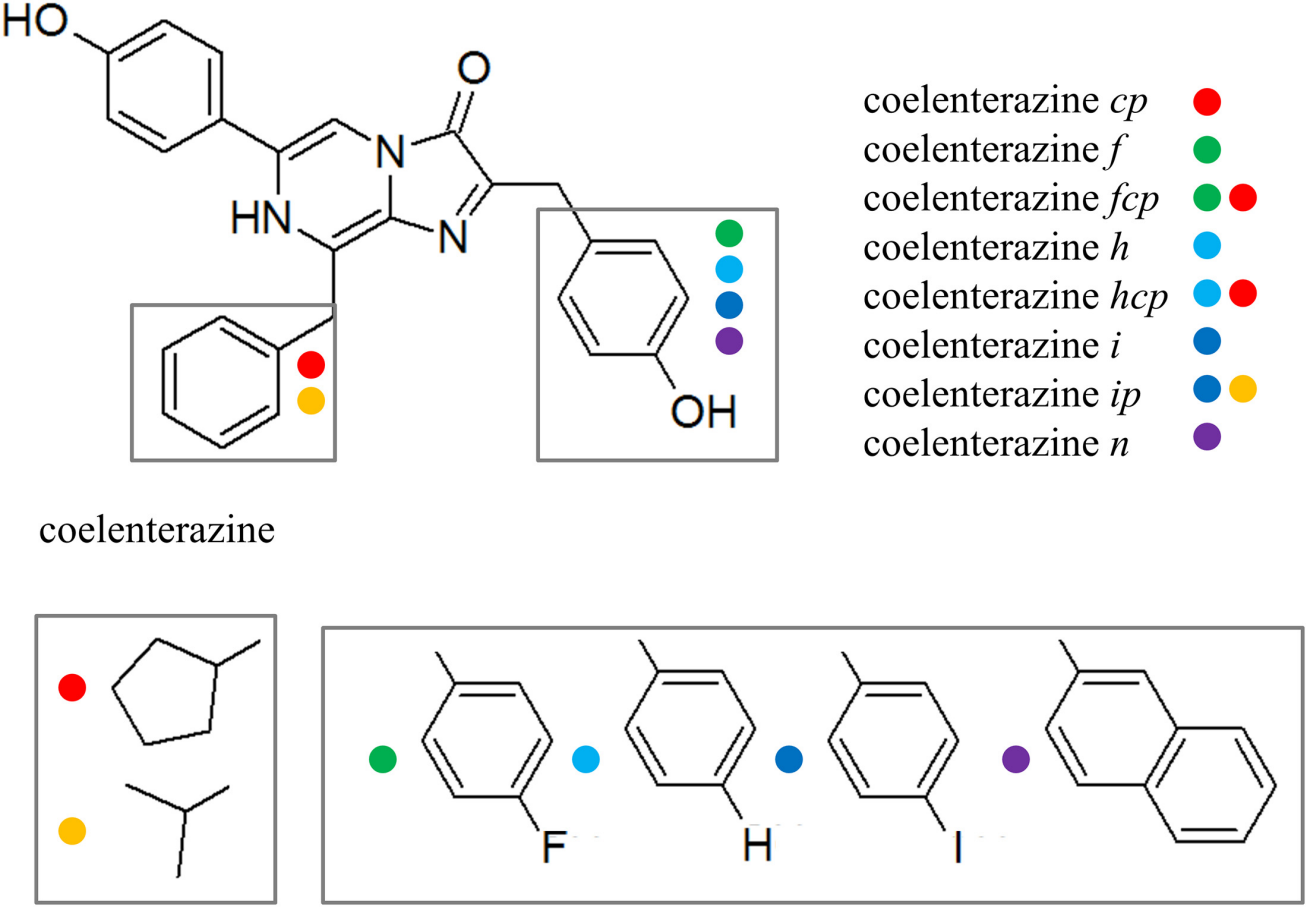
Structures of synthetic coelenterazines. Reprinted from [47] under a CC BY license, with permission from *Open Access Dissertations*, original copyright 2015.

Previous studies have shown that mutations in the protein sequence involving the Tyr82-His16-Trp86 triads using canonical amino acids have a great effect on the spectral characteristics of aequorin while maintaining its bioluminescent activity, most notably a red-shift in the emission wavelength. This led to the selection of the tyrosine at position 82 and tryptophan at position 86 for replacement in a cysteine-free aequorin variant (hereafter “aequorin“), to minimize the absorption of the signal by tissue during *in vivo* studies, such as in calcium imaging and in the previously mentioned deep tissue studies [41, 55]. In this study, the tryptophan residue at position 86 was replaced with four different non-natural amino acids L-4-aminophenylalanine (AminoPhe), L-4-bromophenylalanine (BromoPhe), L-4-iodophenylalanine (IodoPhe), and L-4-methoxyphenylalanine (MethoxyPhe). The position was also substituted simultaneously with the tyrosine at position 82. The variants were then complexed with native coelenterazine and eight synthetic coelenterazines *cp*, *f*, *fcp*, *h*, *hcp*, *i*, *ip*, and *n* (Fig 2).

To test the applicability of these red-shifted aequorin variants for *in vivo* imaging the variant with the greatest red-shifted wavelength and the variant with the longest half-life were each injected introsomally into the eye of a 6–8 week old ICR (CD1) outbred female mouse. The bioluminescence was then triggered by topical addition of coelenterazine and the emission of light detected with a IVIS instrument. Though aequorin has been part of imaging tissues as difficult to access as the deep brain, [56], the eye was chosen for our initial visualization studies because of its characteristics of optical transparency, simple anatomy, well-defined structure, and easy accessibility.

## Results and Discussion

To circumvent the limitations of current luminescent proteins in imaging and expand their use *in vivo*, we have prepared a catalog of mutants of the tunable photoprotein aequorin [8, 26, 46, 57]. We prepared these variants by site-specifically incorporating non-natural amino acids into selected sites of the hydrophobic pocket of aequorin using Amber Suppression [42, 55, 58, 59]. Previous work in our group employing Amber Suppression replaced a tyrosine residue at position 82 by introducing a TAG codon to create functional non-natural variants of aequorin with red-shifted wavelengths of emission and longer bioluminescence half-lives [46]. In the present study, our group individually incorporated four different non-natural amino acids, specifically, L-4-aminophenylalanine (AminoPhe), L-4-bromophenylalanine (BromoPhe), L-4-iodophenylalanine (IodoPhe), and L-4-methoxyphenylalanine (MethoxyPhe), into aequorin at position 86 alone and 82 and 86 together (Fig 1). These non-natural amino acid residues were selected for their hydrophobicity, aromaticity, and their size similarity to tyrosine and tryptophan [42–44]. By complexing them with native coelenterazine and eight synthetic coelenterazine analogs, this work resulted in 72 new bioluminescent aequorin variants; several of these variants had red-shifted wavelengths of emission, and both shorter and longer bioluminescence half-lives were observed as compared to those of native aequorin.

Two plasmid systems were developed to express the aequorin variants. The first system incorporated the non-natural amino acids into position 86 using a pBAD/HisA vector by using well-established molecular biology protocols [60] that was co-transformed with pDULE vectors engineered for each non-natural amino acid procured from the Schultz group [61]. This resulted in a system in which the expression of aequorin is tightly regulated by the *ara*BAD promoter and protein expression is induced by the addition of arabinose to the culture medium. The culture was then spun down and aequorin isolated from the supernatant by acid precipitation. Low-level expression of the L-4-aminophenylalanine aequorin prompted the generation of a second system in which the aequorin gene with a TAG codon at position 86 was inserted into a pET30Xa/LIC plasmid. This construct produced a good yield of the 86 variant aequorin and was used in all subsequent experiments (S1 Fig). The pETXa/LIC also included a His6x tag to aid in purification.

The protein variants were prepared by supplementing the culture media containing the appropriate plasmids with the selected non-natural amino acid and expression was induced with IPTG. The cells were lysed, and the protein variants were isolated and purified using affinity chromatography. Incorporation of the non-natural amino acids was verified by mass spectrometry (S1 Table, S7–S11 Tables) and the resulting variants were complexed with one of nine different coelenterazine analogs: *native*, *cp*, *f*, *fcp*, *h*, *hcp*, *i*, *ip*, or *n*. The resulting aequorin variants were characterized for specific activity, wavelength emission maximum, and bioluminescence emission half-life (S2–S4 Tables). Though not all mutants displayed a marked red-shift and also displayed the loss in specific activity associated with synthetic coelenterazine analogs, several mutant aequorin and synthetic coelenterazine complexes showed suitable emission characteristics.

**Fig 3:**
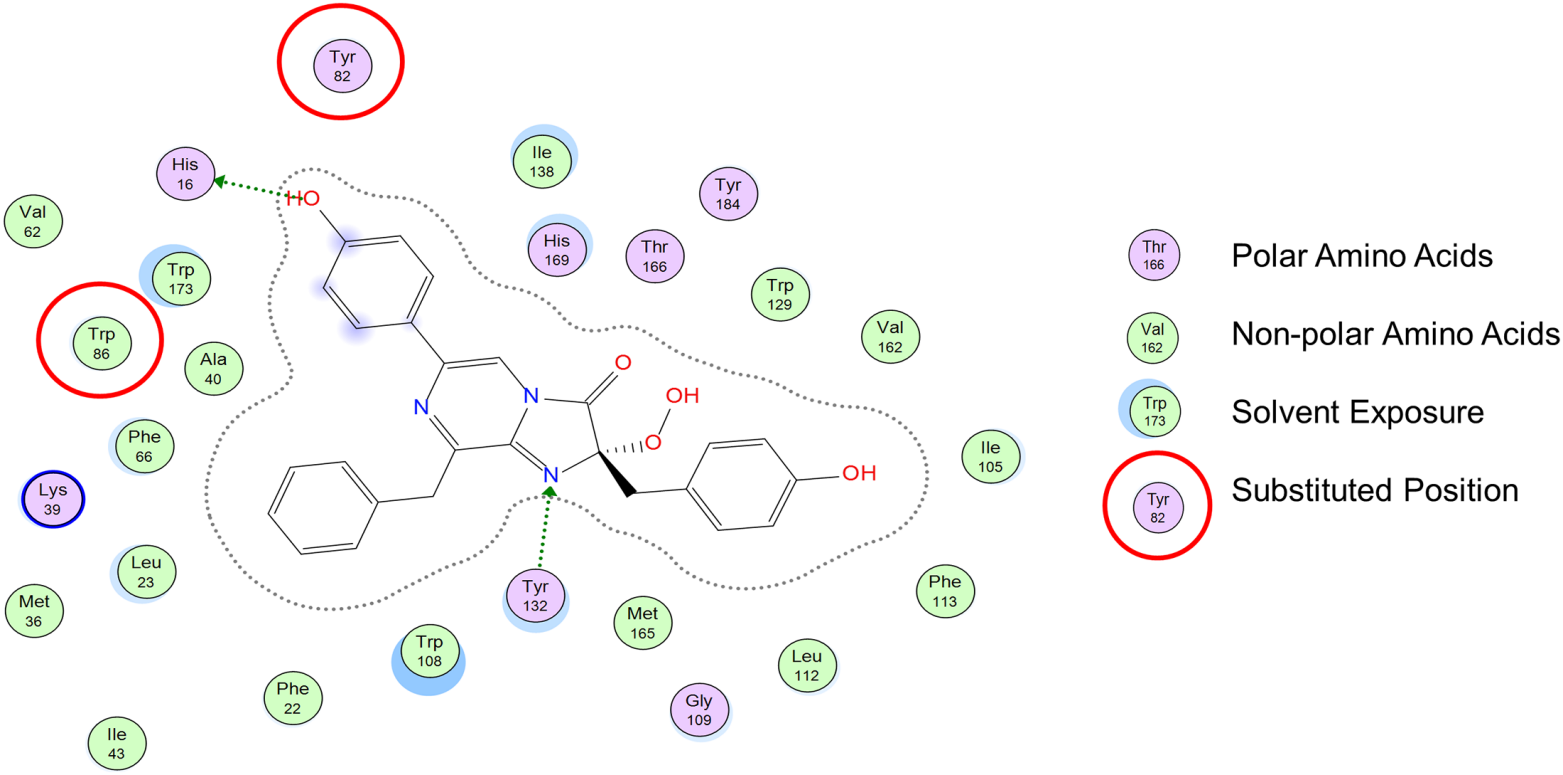
Native coelenterazine in the binding pocket of aequorin. The grey dashed lines represent the Van der Waal surface of the ligand. Green dashed lines with an arrowhead represent an H-bond going from donor to recipient. The structure is based on aequorin’s crystal structure [43](PDB ID: 1EJ3). Reprinted from [47] under a CC BY license, with permission from *Open Access Dissertations*, original copyright 2015.

The data in Table 1 shows the change in the wavelength maximum emission spectra and bioluminescence emission half-lives for selected variants as well as wild-type aequorin. The aequorin variants with single substitutions at position 86 showed longer bioluminescence emission half-lives than those found in our previous study with an aequorin containing a single non-natural incorporation at position 82. Further, the double non-natural incorporation produces variants with a marked red-shifted wavelength of bioluminescence emission [46]. Most significantly, aequorin with L-4-methoxyphenylalanine at positions 82 and 86 complexed with coelenterazine *i* emits maximal bioluminescence at 526 nm, which is the largest red-shift of wavelength of any aequorin variant reported thus far and 11 nm longer than the previous most red-shifted aequorin, namely, L-4-methoxyphenylalanine at position 82 [46]. Fig 3 shows the interactions of the tryptophan residue at position 86 with the coelenterazine molecule. By mutating the Trp86, the stability of the phenol ring moiety associated with the Tyr82-His16-Trp86 triad is affected by the change in the H-bond network. In examining the binding interactions of the amino acids in the binding pocket with the newly prepared variant aequorin with L-4-aminophenylalanine and L-4- methoxyphenylalanine, it is evident that these non-natural amino acids, which have an amino group and methoxy group, respectively, are capable of maintaining a polar interaction with coelenterazine, while L-4-bromophenylalanine and L-4-iodophenylanine are not. We postulate that the loss of the H-bonds that stabilize the luciferin could explain the red-shifted emission wavelengths of the halogenated aequorin variants L-4-bromophenylalanine and L-4-iodophenylanine. Also, the presence of iodine in a molecule can increase the transition probability of an electron in an excited singlet state to an excited triplet state, creating a shift in the emission spectra as well as the elongation of the half-life [62].

**Table 1:** Emission characteristics of selected aequorin variants.

Aequorin and CTZ	Wavelength (nm)	Half-life (s)
Wild-type Aequorin and *Native*	472	0.5
IodoPhe 86 *Native*	499	14.3
IodoPhe 86 *i*	508	38.01
AminoPhe 82&86 *Native*	496	3.57
AminoPhe 82&86 *i*	515	58.68
BromoPhe 82&86 *Native*	500	3.21
BromoPhe 82&86 *i*	514	55.62
IodoPhe 82&86 *Native*	500	4.19
IodoPhe 82&86 *i*	508	64.74
MethoxyPhe 82&86 *Native*	513	3.53
MethoxyPhe 82&86 *i*	526	48.73

**Fig 4:**
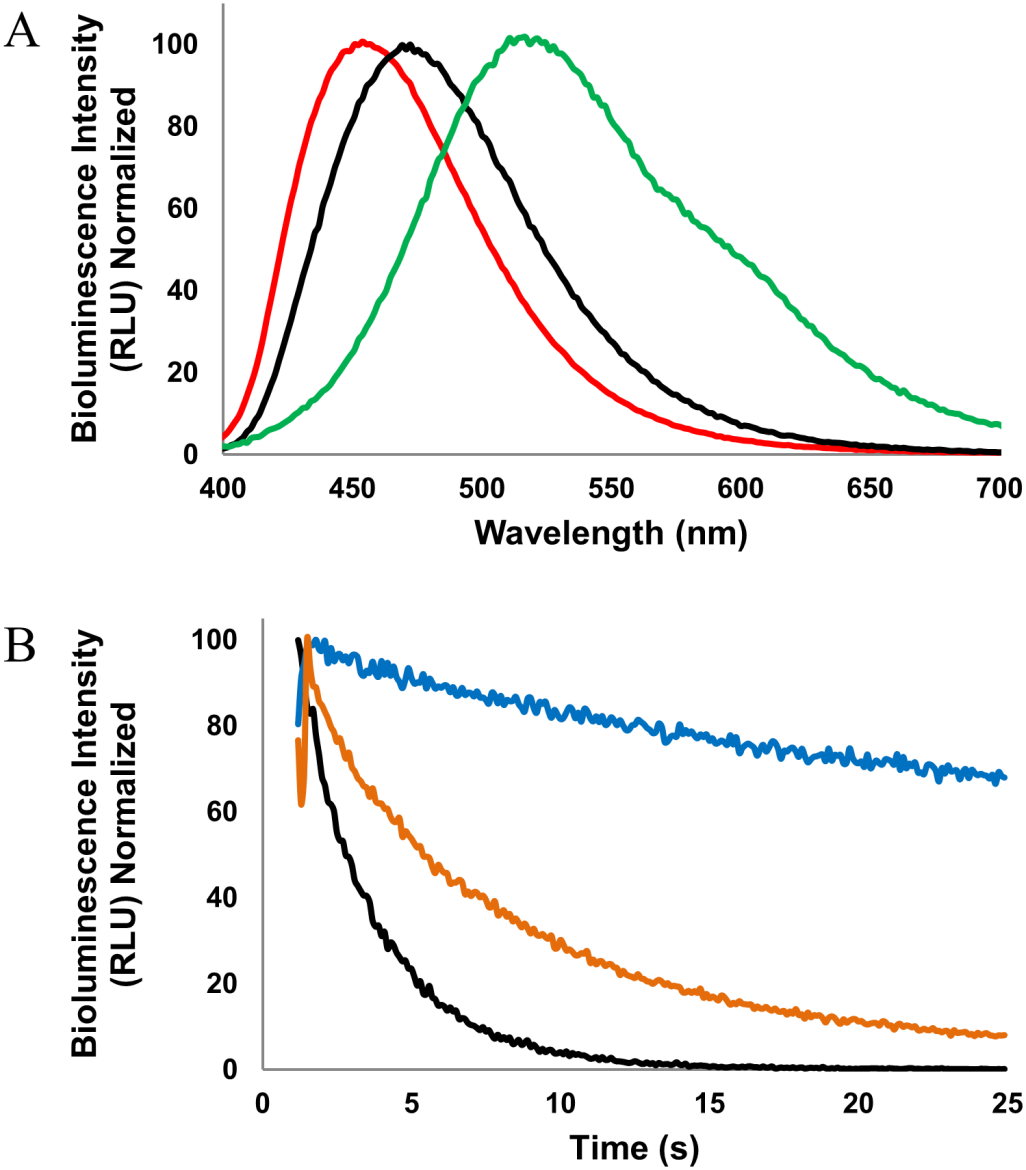
Emission wavelengths and bioluminescence half-lives of selected aequorin variants. (A) Bioluminescence emission spectra of aequorin with L-4-aminophenylalanine at position 86 with coelenterazine *cp* (red), aequorin with native coelenterazine (black), and L-4-methoxyphenylalanine at position 82 and 86 with coelenterazine *i* (green), illustrating the range of emission wavelengths in this study. (B) Half-life bioluminescence decay of aequorin with native coelenterazine (black), L-4-methoxyphenylalanine with coelenterazine *n* (orange), and L-4-iodophenylalanine with coelenterazine *i* (blue), illustrating the range of emission half-lives in this study. Reprinted from [47] under a CC BY license, with permission from *Open Access Dissertations*, original copyright 2015.

While all novel non-natural amino acid-modified aequorin variants show an increase in the half-lives of the bioluminescence emission, the most notable half-life changes are associated with the variants containing coelenterazine *i*. These variants display the longest bioluminescence decay half-lives with an average of approximately 1 minute, while the half-life of aequorin with native coelenterazine is approximately a half second (Table 1). Notably, the aequorin variant with a double substitution of L-4-iodophenylalanine at positions 82 and 86 paired with coelenterazine *i* has the longest bioluminescence half-life emission time reported as well at approximately 60 s, compared to our own previously reported 14 s with a single L-4-methoxyphenylalanine at position 82 paired with coelenterazine *i* (Fig 4) [46].

The increased probability of the transition of the excited electron from singlet state to triplet state due to the heavy atom can explain the differences in half-lives of the coelenterazines that have iodine compared to the ones that do not possess a heavy atom [62]. This trend is also present in the modified aequorins with a L-4-bromophenylalanine substitution, though to a lesser extent. Interestingly, in our previous work, the single substitution of Tyr82 did not display as great an increase in the half-life of the bioluminescence emission as the single substitution at position Trp86, even with coelenterazine *i*, but, in contrast, they did exhibit greater red-shift in emission wavelength. The variants with non-natural amino acid substitutions at both positions, Tyr82 and Trp86, show a mixed influence of both substitutions, with longer emission half-lives as well as greater red-shifted emission spectra. These emission characteristics may allow for multiplex detection by spatial and temporal resolution through the differentiation of the emission wavelength and time [51].

**Fig 5:**
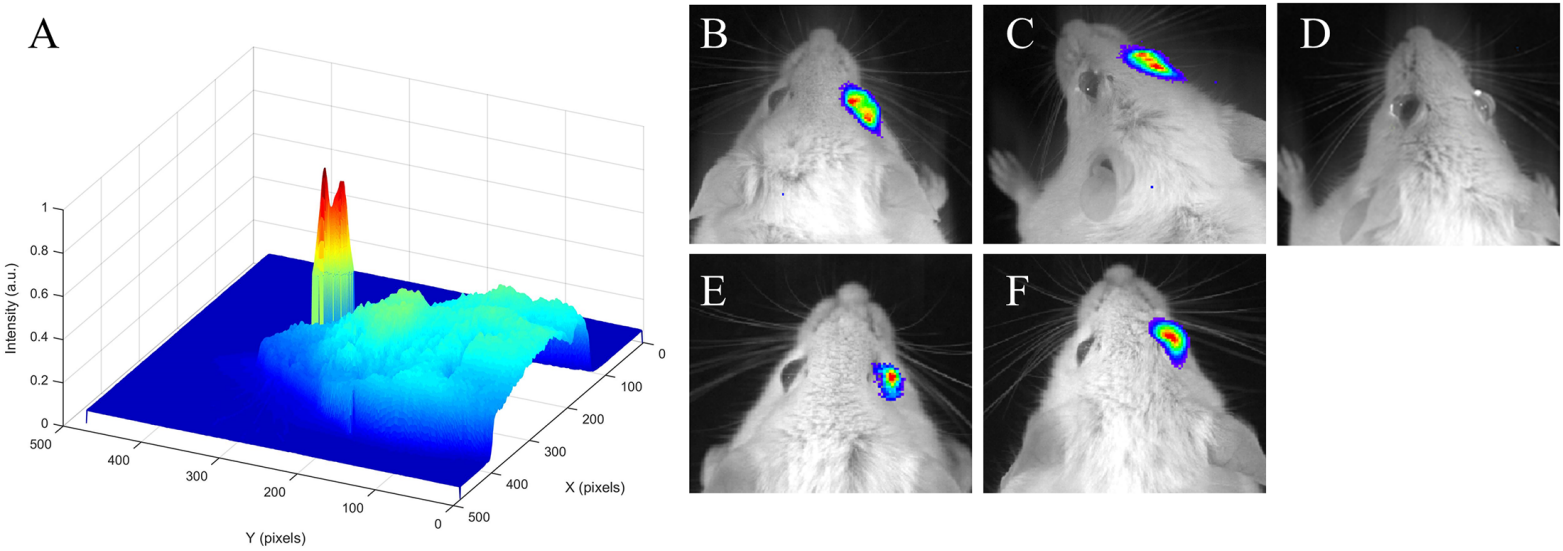
Bioluminescence emission of selected aequorin variants in an *in vivo* mouse model. Mice received a 5 μL intrastromal or antechamber injection in the right eye with variant aequorin and the left eye with HEPES Buffer. (A,B) Aequorin injected intrastromally at a concentration of 2.7 x 10^−5^ M, 30 s exposure. (C) Aequorin with L-4-methoxyphenylalanine at positions 82 and 86. (D) HEPES Buffer only in both eyes. (E) Aequorin injected in the antechamber at a concentration of 3.2 x 10^−5^ M, 60 s exposure. (F) Aequorin with L-4-iodophenylalanine at positions 82 and 86. Images generated with Matlab R2014b and LivingImage 4.4. Reprinted from [47] under a CC BY license, with permission from *Open Access Dissertations*, original copyright 2015.

The feasibility of employing the red-shifted aequorin variants for *in vivo* imaging was explored by injecting the photoproteins into the right eyes (intrastromal as well as antechamber) of anesthetized mice, chosen for their easy accessibility and simple anatomy as well as for the potential to replace fluorescence in eye studies. The variants with L-4-methoxyphenylalanine at positions 82 and 86 and L-4-iodophenylalanine at positions 82 and 86 were chosen for their red-shifted emission wavelength and long bioluminescence half-life, respectively. The left eyes of the mice were only injected with buffers to serve as internal controls. Additionally, mice without aequorin injected into either eye were imaged. Fig 5 shows the imaging experiments performed by intrastromal injection of identical concentrations of aequorin and L-4-methoxyphenylalanine variants. Native coelenterazine was added to the surface of the eye dropwise to turn “on” the light, that is, trigger the emission of the flash of bioluminescence from the photoproteins, which was immediately recorded by the IVIS. External addition of calcium ions was not necessary since the eye already contains an abundance of calcium ions. Fig 5 also shows bioluminescence emission from the aequorin and the L-4-iodophenylalanine variant injected into the antechamber of the eye. The L-4-iodophenylalanine variant was added at a greater concentration due to the lower specific activity exhibited in the *in vitro* studies (S2 Table). The mice were observed for up to 1 1/2 hours after the addition of the coelenterazine and still displayed bioluminescence, attributed to the time necessary for the coelenterazine to diffuse to the aequorin. There was also no bioluminescent signal outside of the eye itself, demonstrating that the aequorin did not diffuse to other areas after injection. These results with *in vivo* imaging suggest that these red-shifted aequorin variants could also be used for imaging studies in other tissues or organs in animals in the future. Bioluminescent resonance energy transfer (BRET) employing aequorin has been demonstrated effective in deep tissue studies, thus suggesting that the aequorin designed, prepared, and employed herein could potentially be translated to applications in deeper tissues [63, 64].

## Conclusions

In conclusion, our data demonstrate that aequorin variants containing non-natural amino acids in place of the tryptophan residue at position 86 and the tyrosine at position 82 showed red-shifted bioluminescence activity when complexed with native coelenterazine. Moreover, the peak emission wavelengths and half-lives of these variants can further be extended by using coelenterazine analogs. Though not all the mutants showed a significant shift and displayed a drop in specific activity associated with coelenterazine analogs, several had shifts beyond 45 nm and were tested *in vivo*. The aequorin variant with L-4-methoxyphenylalanine at positions 82 and 86 with complexed coelenterazine *i* displays a Δ56 nm red-shift in bioluminescence emission wavelength, being 526 nm the greatest red-shift reported thus far for an aequorin variant. Thus, this broadens the realm of applications of aequorin variants as reporter molecules in multiplex applications and *in vivo* imaging. These red-shifted aequorin variants showed excellent performance in imaging experiments when applied to the eye of a mouse, thus demonstrating their potential use in molecular imaging for diagnostics and/or management of disease. In summary, the red-shifted aequorin variants prepared and reported herein demonstrated improved characteristics for *in vivo* imaging with regard to our previously reported photoproteins, broadening the applications of aequorin in biomedical analysis and diagnostics.

## Materials and Methods

### Ethics statement

All animal procedures were performed in compliance with the Association for Research and Vision in Ophthalmology (ARVO) Statement for the use of Animals in Ophthalmic and Vision Research and in accordance with the Guide for the Care and Use of Laboratory Animals (protocol 13–034) published by the National Institutes of Health and approved by the Animal Care and Use Committee of the University of Miami.

### Reagents

The pBAD/His A and Terrific Broth (TB) are from Invitrogen (Carlsbad, CA). The PET-30 Xa/LIC Kit and Xa Factor Cleavage Capture Kit are from Novagen (Madison, WI). NcoI and HindIII restriction endonucleases are from New England Biolabs (Boston, MA). All natural amino acids, and all antibiotics, are from Sigma-Aldrich (St. Louis, MO). LB agar and LB broth are from Fischer Scientific (Fair Lawn, NJ). The coelenterazines are from Gold Biotechnology (St. Louis, MO). The L-4-aminophenylalanine, L-4-bromophenylalanine, L-4-iodophenylalanine, and L-4-methoxyphenylalanine are from Peptech (Burlington, MA).

### Apparatus

Cells were grown in a Thermo-Fisher Scientific orbital shaker incubator at 37°C. Cell cultures were harvested using a Beckman J2-MI centrifuge. The proteins were purified using a BioCad Sprint Perfusion Chromatography System (Perseptive Biosystems, Farmington, MA) using 20 mL DEAE Waters AP-2 Anion Exchange Column (Waters Corporation, Milford, MA). The buffers for the purification of the protein were 30 mM Tris-HCl, pH 7.5, containing 2 mM EDTA (Buffer A) and 30 mM Tris-HCl, pH 7.5, containing 2 mM EDTA, and 1 M NaCl (Buffer B). Diafiltration was performed using a tangential flow separation module with hollow fiber filter membrane (Spectrum Labs, Rancho Dominguez, CA) using 30 mM Tris-HCl, pH 7.5, containing 2 mM EDTA. Ni-NTA Agarose beads are from Qiagen (Venlo, Netherlands). Amicon Ultra-15 Centrifugal Filter Units are from EMD (Billerica, MA). Purity of the proteins was confirmed by the appearance of a single band after sodium dodecyl sulfate-polyacrylamide gel electrophoresis (SDS-PAGE) using a Novex Mini-cell apparatus from Invitrogen and staining with Coomassie Brilliant Blue. Aequorin activity was measured using an Optocomp I luminometer (MGM Biomedical Hamden, CT). The emission spectra of the aequorins were determined using a custom made SpectroScan instrument (ScienceWares, Falmouth, MA), which is capable of obtaining spectra from flash reactions of luminescent samples that emit in the 400–700 nm range. Half-life scans were taken using a Polarstar Optimax 96 well microplate reading luminometer (BMG Labtech, Ortenberg, Germany).

### Construction of the pDULE-pBADHisA-AEQTAG86 expression strains

Four pDULE vectors which allow for the site-specific incorporation of four different non-natural amino acids were obtained from Dr. Peter Schultz (Scripps Research Institute, La Jolla CA) and Dr. Ryan Mehl (Franklyn and Marshall College, Lancaster, PA) [61]. Each of these pDULE plasmids coded for a tRNA_CUA_ and tRNA_CUA_-synthetase specific for the TAG codon and a single non-natural amino acid, either L-4-aminophenylalanine, L-4-bromophenylalanine, L-4-iodophenylalanine, or L-4-methoxyphenylalanine. These plasmids were transformed into *Escherichia coli* DH10B cells and transformants were selected by plating on LB agar medium containing ampicillin (100 μg/mL) and tetracycline (12 μg/mL). The presence of the pDULE plasmid was confirmed by plasmid isolation, restriction enzyme digestion and DNA gel electrophoresis.

### Construction of the pDULE-pET30AEQ8286TAG expression strains

The four pDULE plasmids were transformed into *E*. *coli* DH10B cells with pET30AEQ8286TAG and transformants were selected by plating on LB agar medium containing tetracycline (12 μg/mL) and kanamycin (35 μg/mL). The presence of the pDULE plasmid was confirmed by plasmid isolation, restriction enzyme digestion and DNA gel electrophoresis.

pBADAEQ86TAG was prepared by inserting the AEQTAG86 gene with the ompA leader sequence attached into pBAD/HisA. The pIN4AEQ86TAG vector containing the cysteine-free aequorin gene fused to the ompA leader sequence was used as a template. The primers were designed so the resulting DNA sequence contained an NcoI site at the 5’ end and a HindIII site at the 3’ end.

The following primers were used for the cloning of ompA:AEQ86TAG fusion into pBAD/HisA vector (restriction sites for cloning are underlined).

#### AEQforpBAD

5'-CCATGGGTATGAAAAAGACAGCTATCGCGATTGC-3’

#### AEQrevpBAD

5'AAGCTTAGGGGACAGCTCCACCGTAGAGCTTTTCGGAAGCAGGATCCATTGTGTAC-3’

The resulting PCR product was gel purified and cloned into the pCR^®^II-TOPO^®^ vector by using the TOPO TA cloning kit (Invitrogen, Carlsbad, CA). The plasmid from the TA clone was then isolated and sequenced to confirm the presence of the insert. The ompA:AEQ86TAG insert from the TOPO TA clone was then ligated into the NcoI and HindIII sites of pBAD/His A to create pBADHisA-AEQ86TAG.

The AEQTAG86 gene was also cloned into a pET30 Xa/LIC Vector using primers designed according to the manufacturer’s instructions. pET30AEQ8286TAG was then prepared by using a pair of primers to create a site-specific mutation consisting of a TAG at position 82 (underlined).

#### Oligo HPLCTAG8286for

5'-GGAAACTGATTGGCCTGCATAGATTGAAGG-3’

#### HPLCTAG8286rev

5'-CCTTCAATCTATGCAGGCCAATCAGTTTCC-3’

pBADHisA-AEQ86TAG and pET30AEQ8286TAG vectors were transformed into chemically competent *E*. *coli* DH10B cells that already contained the pDULE plasmids to produce four different strains, each specific for the incorporation one of the four non-natural amino acids mentioned above. Selection for transformants was performed by plating on LB agar medium containing both ampicillin (100 μg/mL) kanamycin (35 μg/mL) for cells containing pBADHisA-AEQ86TAG and tetracycline (12 μg/mL) and kanamycin (35 μg/mL) for cells containing pET30AEQ8286TAG.

### Expression and isolation of aequorin variants from pBADHisA-AEQ86TAG

The cells harboring the pDULE systems with pBADHisA-AEQ86TAG vector were grown in 25 mL of LB broth containing ampicillin (100 μg/mL) and tetracycline (12 μg/mL). All cultures, unless specified otherwise, were grown overnight at 37°C with shaking at 250 rpm. These overnight cultures were used to inoculate 500 mL of LB broth containing the appropriate antibiotics and the cultures were grown at 37°C at 250 rpm to an OD_600_ between 0.4–0.6. The corresponding non-natural amino acid was then added to a final concentration of 1 mM. The culture was allowed to grow for 1 hour and then induced with arabinose (0.2% final concentration) overnight.

The culture was centrifuged at 15,300 xg for 20 min and the pellet discarded. The supernatant was acid precipitated using 12 M HCl, then spun again. The pellet was resuspended in Tris-HCl, pH 7.5, containing 2 mM EDTA.

### Expression and isolation of aequorin variants from pET30AEQ8286TAG

The cells were grown in 25 mL of Terrific broth (TB) containing tetracycline (12 μg /mL) and kanamycin (35 μg/mL) overnight at 37°C at 250 rpm. The overnight cultures were used to inoculate 500 mL of TB broth containing the same antibiotics and the cultures were grown at 37°C at 250 rpm. After growing to an OD_600_ between 0.4–0.6, the corresponding non-natural amino acid was added to a final concentration of 1 mM. The culture was allowed to grow for 1 hour and then induced with 1 mM IPTG and grown overnight.

The culture was centrifuged at 15,300 xg for 20 min and the pellet was boiled for 5 min in Native Purification Buffer (50 mM NaH_2_PO_4_, 0.5 M NaCl, 1 mM Imidazole, pH = 8.0). The boiled pellet was centrifuged again and the supernatant added to 1 mL of suspended Ni-NTA agarose beads and rotated on a Mini Labroller (Labnet, Woodridge, NJ) at room temperature for 2 hours. The protein was eluted off the column using a PBS Buffer with 20 mM imidazole at pH = 8.0 and digested with Xa factor according to manufacturer’s instructions until all the His tag was cleaved from the protein. The Xa factor was removed with Xa factor Agarose capture beads. The cleaved aequorin was then concentrated using Amicon Ultra spin columns.

### Determination of activities of aequorin variants

Aequorin was diluted with 30 mM Tris-HCl, pH 7.5, containing 2 mM EDTA, to 1x10^-7^ M and charged with 1x10^-4^ M coelenterazine overnight. Aequorin activity was triggered by injecting 100 μL of 100 mM Tris-HCl, pH 7.5, containing 100 mM CaCl_2_. Bioluminescence intensity was measured at 0.1 s intervals for up to 6 s on an Optocomp I luminometer (MGM Biomedical Hamden, CT). Units are in Relative Light Units (RLU) per mole. N = 3 or more, standard deviation is 5% or less.

### Emission spectra of aequorin variants

A 1 x 10^−6^ M sample of each aequorin was charged by incubating overnight at 4°C with 1x10^-4^ M of each of the different coelenterazines and 150 μL of each charged aequorin was pipetted into a 96-well microtiter plate and following injection of 100 μL of 100 mM Tris-HCl, pH 7.5, containing 100 mM CaCl_2_, the emission spectra was collected for 10 s over a scanning range of 400–700 nm in 1.5 nm increments on a custom made SpectroScan instrument (ScienceWares, Falmouth, MA). N = 3 or more, standard deviation is 5% or less.

### Half-life determination of aequorin variants

A 1x10^-6^ M sample of each aequorin variant was charged overnight at 4°C with the 1x10^-4^ M of the coelenterazine analogue used during the collection of emission spectra. A Polarstar Optimax 96 well microplate luminometer (BMG Labtech, Ortenberg, Germany) was utilized for the half-life measurements. Filters were not required. The bioluminescence signal of a 50 μL sample was collected between 30 s to 150 s, depending on the expected half-life of the aequorin analogue, following the injection of 100 μL of triggering buffer (100 mM Tris-HCl, pH 7.5, containing 100 mM CaCl_2_). The mean bioluminescence decay spectra was fit with an exponential decay equation using GraphPad Prism 5.0 (GraphPad Software, San Diego, CA), and an equation for first order decay kinetics was used to calculate the bioluminescence half-life of each aequorin-coelenterazine pair. N = 3 or more, standard deviation is 5% or less.

### Mass spectrometry

Proteins were run on SDS-PAGE and cut out of the stained gels with Coomassie Blue. Gel pieces were digested with trypsin, and an LC-ESI-MS-MS was performed using a ThermoFinnigan LTQ. Resulting MS-MS spectra were compared with proteins in the Swiss-Prot database using the X!Tandem search engine to look for the presence of the substitution of the non-natural amino acid at and only at the target positions.

### *In vivo* imaging

The aequorin variants were dissolved in a HEPES Buffer (10 mM HEPES, 0.15 M NaCl at pH = 7.4) at concentrations from 9.1 x 10^−6^ M to 4.5 x 10^−5^ M. All mice were anesthetized with a 100 μL ketamine/xylazine (1.5 mg/0.3 mg) by intraperitoneal injection before the injection of the sample. The aequorin samples were injected into one eye of six mice via intrastromal injection in 5 μL volumes, or into the antechamber at a volume of 10 μL, of each of the ICR (CD1) outbred female mice (6–8 weeks old, purchased from Taconic (Germantown, NY)). The same volumes of plain HEPES were injected into the other eye to serve as an internal control. Native coelenterazine was diluted in PBS buffer to a volume of 2.3 x 10^−4^ M and dropped onto the surface of each eye in 1 μL volumes. The mice were then placed in a Caliper/Xenogen IVIS^®^ SPECTRUM (Caliper, Hopkinton, MA) in the IVIS Small Animal Imaging Facility at the Oncogenmonics Core Facility, Miller School of Medicine, with oxygen flowing into the imaging chamber. Images were taken over 30 s to 60 s.

## Supporting Information

S1 Fig Plasmids for expression of aequorin variant proteins. (A) pBAD-based plasmid inducible by arabinose for the expression of variant aequorin with a singular amber mutation at position 86. (B) pET30-based plasmid inducible by IPTG for the expression of aequorin with an amber mutation at positions 82 and 86 and the sequence encoding for a His6xtag at the N-terminus.
(DOC)

S2 Fig SDS Gel Showing Purified Aequorin and Aequorin with L-4-iodophenylalanine at position 82 and 86. (DOC)

S1 Table Mass spectrometry data for the aequorin mutants. (A) Mass spectrometry for a single substitution. Tryptophan 86 is located at the end of the peptide fragment (W*). (B) Mass spectrometry data for a double substitution. Tyrosine 82 is located near the middle of the fragment (Y*).
(DOC)

S2 Table Specific activity of aequorin variants: (A) Single substitution at position 86 and (B) Double substitution aequorin variants at positions 82 and 86. (DOC)

S3 Table Emission wavelengths of aequorin variants. (A) Single substitution at position 86 and (B) Double substitutions at 82 and 86 in nm. N = 3 or more, standard deviation is 5% or less.
(DOC)

S4 Table Emission half-lives of aequorin variants. (A) Single substitution at position 86 and (B) Double substitutions at 82 and 86. The half-life values of the bioluminescence are in s. N = 3 or more, standard deviation is 5% or less.
(DOC)

S5 Table Mass Spectrometry Data for AminoPhe86AEQ. (DOC)

S6 Table Mass Spectrometry Data for BromoPhe86AEQ. (DOC)

S7 Table Mass Spectrometry Data for IodoPhe86AEQ. (DOC)

S8 Table Mass Spectrometry Data for MethoxyPhe86AEQ. (DOC)

S9 Table Mass Spectrometry Data for BromoPhe8286AEQ. (DOC)

S10 Table Mass Spectrometry Data for IodoPhe8286AEQ. (DOC)

S11 Table Mass Spectrometry Data for MethoxyPhe8286AEQ. (DOC)
