# Supplementary material for: Red-Shifted Aequorin Variants Incorporating Non-Canonical Amino Acids: Applications in In Vivo Imaging
Source: PLoS One. 2016 Jul 1;11(7):e0158579. doi: 10.1371/journal.pone.0158579 (PMC4930207; doi:10.1371/journal.pone.0158579)
Supplement: S1 Fig — (A) pBAD-based plasmid inducible by arabinose for the expression of variant aequorin with a singular amber mutation at position 86. (B) pET30-based plasmid inducible by IPTG for the expression of aequorin with an amber mutation at positions 82 and 86 and the sequence encoding for a His6xtag at the N-terminus. (DOC) [file pone.0158579.s001.doc]

# Supplementary Information

# Red-Shifted Aequorin Variants Incorporating Non-Canonical Amino Acids. Applications in *In Vivo* Imaging

Kristen Grinstead, Laura Rowe, C. Mark Ensor, Emre Dikici, Jean-Marc Zingg, and Sylvia Daunert


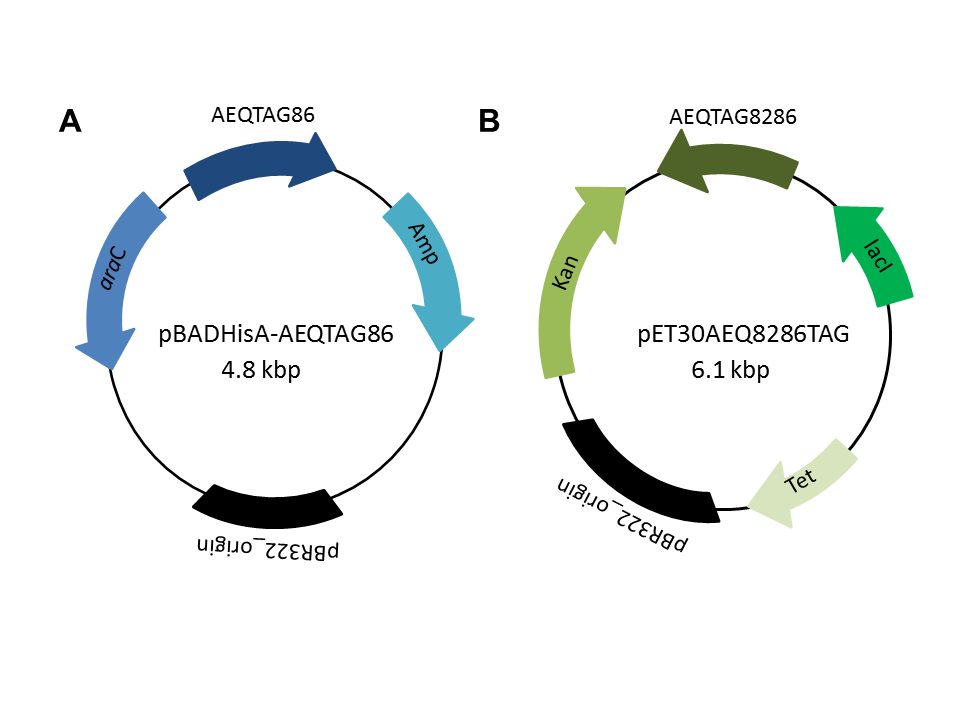


S1 Fig. Plasmids for expression of aequorin variant proteins. (A) pBAD-based plasmid inducible by arabinose for the expression of variant aequorin with a singular amber mutation at position 86. (B) pET30-based plasmid inducible by IPTG for the expression of aequorin with an amber mutation at positions 82 and 86 and the sequence encoding for a His6xtag at the N-terminus.
