# Supplementary material for: Red-Shifted Aequorin Variants Incorporating Non-Canonical Amino Acids: Applications in In Vivo Imaging
Source: PLoS One. 2016 Jul 1;11(7):e0158579. doi: 10.1371/journal.pone.0158579 (PMC4930207; doi:10.1371/journal.pone.0158579)
Supplement: S1 Table — (A) Mass spectrometry for a single substitution. Tryptophan 86 is located at the end of the peptide fragment (W*). (B) Mass spectrometry data for a double substitution. Tyrosine 82 is located near the middle of the fragment (Y*). (DOC) [file pone.0158579.s003.doc]

# Supplementary Information

# Red-Shifted Aequorin Variants Incorporating Non-Canonical Amino Acids. Applications in *In Vivo* Imaging

Kristen Grinstead, Laura Rowe, C. Mark Ensor, Emre Dikici, Jean-Marc Zingg, and Sylvia Daunert

| Aequorin | Peptide Fragment | Aequorin Mass | Fragment Mass | Delta Mass |
| --- | --- | --- | --- | --- |
| AminoPhe | YGVETDWPAYIEGW*K | 1813.96 | 1789.94 | -23 |
| BromoPhe | YGVETDWPAYIEGW*K | 1813.96 | 1851.54 | +38 |
| IodoPhe | YGVETDWPAYIEGW*K | 1813.96 | 1899.72 | +86 |
| MethoxyPhe | YGVETDWPAYIEGW*K | 1813.96 | 1804.84 | -9 |

**A**

| Aequorin | Peptide Fragment | Aequorin Mass | Fragment Mass | Delta Mass |
| --- | --- | --- | --- | --- |
| AminoPhe | YGVETDWPAY*IEGW*K | 1813.96 | 1788.94 | -25 |
| BromoPhe | YGVETDWPAY*IEGW*K | 1813.96 | 1916.72 | +103 |
| IodoPhe | YGVETDWPAY*IEGW*K | 1813.96 | 2010.72 | +197 |
| MethoxyPhe | YGVETDWPAY*IEGW*K | 1813.96 | 1818.94 | -5 |

**B**

S1 Table. Mass spectrometry data for the aequorin mutants. (A) Mass spectrometry for a single substitution. Tryptophan 86 is located at the end of the peptide fragment (W*). (B) Mass spectrometry data for a double substitution. Tyrosine 82 is located near the middle of the fragment (Y*).

sition 82 and 86.
