# Supplementary material for: Red-Shifted Aequorin Variants Incorporating Non-Canonical Amino Acids: Applications in In Vivo Imaging
Source: PLoS One. 2016 Jul 1;11(7):e0158579. doi: 10.1371/journal.pone.0158579 (PMC4930207; doi:10.1371/journal.pone.0158579)
Supplement: S2 Table — (DOC) [file pone.0158579.s004.doc]

# Supplementary Information

# Red-Shifted Aequorin Variants Incorporating Non-Canonical Amino Acids. Applications in *In Vivo* Imaging

Kristen Grinstead, Laura Rowe, C. Mark Ensor, Emre Dikici, Jean-Marc Zingg, and Sylvia Daunert

**A**

| Aequorin | CTZ  native | *cp* | *f* | *fcp* | *h* | *hcp* | *i* | *ip* | *n* |
| --- | --- | --- | --- | --- | --- | --- | --- | --- | --- |
| Cysteine-free | 9.7x10^19^ | 5.9x10^19^ | 3.6x10^19^ | 3.9x10^18^ | 3.5x10^19^ | 9.9x10^18^ | 4.4x10^18^ | 2.6x10^19^ | 3.7x10^18^ |
| AminoPhe | 1.1x10^16^ | 7.9x10^15^ | 1.7x10^16^ | 5.5x10^14^ | 7.8x10^15^ | 3.0x10^15^ | 1.5x10^15^ | 8.2x10^14^ | 7.0x10^14^ |
| BromoPhe | 4.0x10^17^ | 1.9x10^18^ | 3.6x10^17^ | 1.2x10^17^ | 4.3x10^17^ | 1.9x10^17^ | 2.9x10^17^ | 1.2x10^17^ | 2.0x10^16^ |
| IodoPhe | 9.7x10^15^ | 1.4x10^16^ | 2.2x10^16^ | 6.8x10^15^ | 1.5x10^16^ | 6.7x10^15^ | 2.3x10^15^ | 8.0x10^15^ | 8.6x10^14^ |
| MethoxyPhe | 1.1x10^17^ | 3.6x10^17^ | 1.3x10^16^ | 7.6x10^15^ | 4.9x10^16^ | 2.5x10^16^ | 2.1x10^15^ | 7.0x10^14^ | 7.5x10^14^ |

| Aequorin | CTZ  native | *cp* | | *f* | | *fcp* | *h* | | *hcp* | | | *i* | | *ip* | *n* |
| --- | --- | --- | --- | --- | --- | --- | --- | --- | --- | --- | --- | --- | --- | --- | --- |
| Cysteine-free | 9.7x10^19^ | 5.9x10^19^ | 3.6x10^19^ | | 3.9x10^18^ | | | 3.5x10^19^ | | 9.9x10^18^ | 4.4x10^18^ | | 2.6x10^19^ | | 3.7x10^18^ |
| AminoPhe | 2.8x10^14^ | 2.5x10^14^ | 5.6x10^14^ | | 1.5x10^14^ | | | 3.8x10^14^ | | 1.9x10^14^ | 1.6x10^14^ | | 1.0x10^14^ | | 1.5x10^14^ |
| BromoPhe | 4.5x10^12^ | 3.1x10^13^ | 1.3x10^13^ | | 4.9x10^12^ | | | 1.7x10^12^ | | 1.9x10^12^ | 3.1x10^12^ | | 1.5x10^12^ | | 1.6x10^12^ |
| IodoPhe | 1.2x10^13^ | 2.8x10^12^ | 3.4x10^12^ | | 1.0x10^12^ | | | 4.6x10^12^ | | 1.0x10^12^ | 6.8x10^11^ | | 2.1x10^11^ | | 9.5x10^11^ |
| MethoxyPhe | 8.0x10^13^ | 4.8x10^13^ | 6.5x10^13^ | | 4.6x10^13^ | | | 5.1x10^13^ | | 2.2x10^13^ | 6.2x10^13^ | | 3.4x10^13^ | | 1.4x10^14^ |

**B**

S2 Table. Specific activity of aequorin variants. (A) Single substitution at position 86 and (B) Double substitution aequorin variants at positions 82 and 86. Units are in Relative Light Units (RLU) per mole. N=3 or more, standard deviation is 5% or less.
