# Supplementary material for: Red-Shifted Aequorin Variants Incorporating Non-Canonical Amino Acids: Applications in In Vivo Imaging
Source: PLoS One. 2016 Jul 1;11(7):e0158579. doi: 10.1371/journal.pone.0158579 (PMC4930207; doi:10.1371/journal.pone.0158579)
Supplement: S3 Table — (A) Single substitution at position 86 and (B) Double substitutions at 82 and 86 in nm. N = 3 or more, standard deviation is 5% or less. (DOC) [file pone.0158579.s005.doc]

# Supplementary Information

# Red-Shifted Aequorin Variants Incorporating Non-Canonical Amino Acids. Applications in *In Vivo* Imaging

Kristen Grinstead, Laura Rowe, C. Mark Ensor, Emre Dikici, Jean-Marc Zingg, and Sylvia Daunert

**A**

| Aequorin | CTZ  native | *cp* | *f* | *fcp* | *h* | *hcp* | *i* | *ip* | *n* |
| --- | --- | --- | --- | --- | --- | --- | --- | --- | --- |
| Cysteine-free | 472 | 454 | 480 | 463 | 472 | 454 | 484 | 454 | 475 |
| AminoPhe | 496±2.7 | 487±4.4 | 502±4.6 | 486±2.8 | 494±1.5 | 480±2.6 | 515±4.9 | 479±2.1 | 509±4.6 |
| BromoPhe | 500±3.5 | 477±3.4 | 507±1.0 | 483±4.7 | 503±1.0 | 481±3.2 | 514±4.7 | 476±3.8 | 518±3.6 |
| IodoPhe | 503±5.0 | 492±1.5 | 457±2.0 | 496±4.5 | 506±3.5 | 483±4.9 | 508±4.9 | 492±4.4 | 494±4.4 |
| MethoxyPhe | 513±4.3 | 490±1.9 | 522±2.5 | 495±4.4 | 513±0.6 | 478±3.2 | 526±3.7 | 482±3.3 | 519±3.8 |

**B**

S3 Table. Emission wavelengths of aequorin variants. (A) Single substitution at position 86 and (B) Double substitutions at 82 and 86 in nm. N=3 or more, standard deviation is 5% or less.
