# Supplementary material for: Red-Shifted Aequorin Variants Incorporating Non-Canonical Amino Acids: Applications in In Vivo Imaging
Source: PLoS One. 2016 Jul 1;11(7):e0158579. doi: 10.1371/journal.pone.0158579 (PMC4930207; doi:10.1371/journal.pone.0158579)
Supplement: S4 Table — (A) Single substitution at position 86 and (B) Double substitutions at 82 and 86. The half-life values of the bioluminescence are in s. N = 3 or more, standard deviation is 5% or less. (DOC) [file pone.0158579.s006.doc]

# Supplementary Information

# Red-Shifted Aequorin Variants Incorporating Non-Canonical Amino Acids. Applications in *In Vivo* Imaging

Kristen Grinstead, Laura Rowe, C. Mark Ensor, Emre Dikici, Jean-Marc Zingg, and Sylvia Daunert

| Aequorin | CTZ  native | *cp* | *f* | *fcp* | *h* | *hcp* | *i* | *ip* | *n* |
| --- | --- | --- | --- | --- | --- | --- | --- | --- | --- |
| Cysteine-free | 0.59 | 0.17 | 0.59 | 0.37 | 0.23 | 0.13 | 14.3 | 0.67 | 1.66 |
| AminoPhe | 4.9±0.83 | 0.61±0.14 | 2.01±1.12 | 0.96±0.36 | 1.20±1.00 | 0.47±0.31 | 33.28±10.8 | 2.67±1.78 | 8.37±3.56 |
| BromoPhe | 2.83±0.49 | 0.51±0.52 | 2.21±0.14 | 1.0±0.18 | 0.64±0.10 | 0.34±0.09 | 22.51±7.92 | 2.64±0.81 | 8.51±3.81 |
| IodoPhe | 4.49±1.67 | 0.70±0.67 | 2.09±2.1 | 1.04±0.86 | 0.79±1.61 | 0.50±0.78 | 38.01±12.4 | 2.37±3.6 | 1.68±2.9 |
| MethoxyPhe | 9.56±0.27 | 3.71±0.19 | 10.34±0.24 | 1.32±0.15 | 11.66±0.12 | 0.52±0.05 | 30.88±21.84 | 2.97±3.77 | 8.83±3.50 |

A

| Aequorin | CTZ  native | *cp* | *f* | *fcp* | *h* | *hcp* | *i* | *ip* | *n* |
| --- | --- | --- | --- | --- | --- | --- | --- | --- | --- |
| Cysteine-free | 0.59 | 0.17 | 0.59 | 0.37 | 0.23 | 0.13 | 14.3 | 0.67 | 1.66 |
| AminoPhe | 3.37±2.24 | 0.77±0.09 | 2.68±0.56 | 1.40±0.22 | 1.20±.57 | 0.56±0.31 | 58.68±7.25 | 4.20±1.77 | 8.37±0.46 |
| BromoPhe | 3.21±1.3 | 0.94±0.08 | 2.57±0.64 | 1.01±0.52 | 0.68±0.09 | 0.36±0.11 | 55.62±1.68 | 3.50±0.63 | 8.04±4.20 |
| IodoPhe | 4.19±1.44 | 0.87±0.13 | 3.67±0.64 | 1.26±0.16 | 1.77±0.13 | 0.94±0.12 | 64.74±9.01 | 5.38±0.48 | 8.42±0.84 |
| MethoxyPhe | 3.53±1.61 | 0.82±1.81 | 3.79±2.82 | 1.35±0.39 | 0.81±5.12 | 0.42±0.16 | 48.73±0.61 | 5.39±1.14 | 9.48±0.78 |

**B**

S4 Table. Emission half-lives of aequorin variants. (A) Single substitution at position 86 and (B) Double substitutions at 82 and 86. The half-life values of the bioluminescence are in s. N=3 or more, standard deviation is 5% or less.
