# Supplementary material for: Red-Shifted Aequorin Variants Incorporating Non-Canonical Amino Acids: Applications in In Vivo Imaging
Source: PLoS One. 2016 Jul 1;11(7):e0158579. doi: 10.1371/journal.pone.0158579 (PMC4930207; doi:10.1371/journal.pone.0158579)
Supplement: S5 Table — (DOC) [file pone.0158579.s007.doc]

# Supplementary Information

# Red-Shifted Aequorin Variants Incorporating Non-Canonical Amino Acids. Applications in *In Vivo* Imaging

Kristen Grinstead, Laura Rowe, C. Mark Ensor, Emre Dikici, Jean-Marc Zingg, and Sylvia Daunert

Sequence Mass. 22,271

AA Coverage. 15 of 189 (7.9%)

Mass Coverage. 1813.9 of 22,271 (8.1%)

VKLTSDFDNP RWIGRHKHMF NFLDVNHNGK ISLDEMVYKA SDIVINNLGA

TPEQAKRHKD AVEAFFGGAG MKYGVETDWP AYIEGWKKLA TDELEKYAKN

EPTLIRIWGD ALFDIVDKDQ NGAITLDEWK AYTKAAGIIQ SSEDCEETFR VCDIDESGQL DVDEMTRQHL GFWYTMDPAC EKLYGGAVP

YGVETDWPAYIEGW*K

# S5 Table. Mass Spectrometry Data for AminoPhe86AEQ
